# Islet amyloid in recent-onset type 1 diabetes—the DiViD study

**DOI:** 10.1080/03009734.2017.1359219

**Published:** 2017-08-17

**Authors:** Gunilla T. Westermark, Lars Krogvold, Knut Dahl-Jørgensen, Johnny Ludvigsson

**Affiliations:** Department of Medical Cell Biology, Uppsala University, Uppsala, Sweden;; Division of Paediatric and Adolescent Medicine, Oslo University Hospital, Oslo, Norway;; Faculty of Medicine, University of Oslo, Oslo, Norway;; Department of Clinical and Experimental Medicine, Division of Pediatrics and Diabetes Research Centre, Linköping University Hospital, Linköping, Sweden

Dear Editor,

Type 1 diabetes is characterized by an immune-mediated destruction of the beta-cells, associated with autoantibodies against islet antigens developing years ahead of the clinical manifestation. However, the disease is heterogeneous, and its pathogenesis is far from fully understood. Besides focus on the importance of virus, hygiene, early nutrition, gut microbiome, etc., one hypothesis is that factors leading to beta-cell stress, usually associated with type 2 diabetes, also might contribute to development of type 1 diabetes (1).

Islet amyloid is to a varying degree present in almost all individuals with type 2 diabetes, and the beta-cell product islet amyloid polypeptide (IAPP) is the primary constituent of the islet amyloid. IAPP is a member of the calcitonin gene family, and the molecule is signalling through the calcitonin receptor when in combination with receptor activity modifying peptide 1 or 3 (RAMP 1 or RAMP 3), all expressed by beta-cells. The exact biological function of the IAPP remains to be determined, but IAPP is released together with insulin and is involved in regulation of glucose homeostasis (2). The mature IAPP molecule is structurally unfolded, and, *in vitro*, IAPP has a high tendency to aggregate (3). Formation of cytotoxic oligomers is linked to cell death, and IAPP aggregation has been shown to activate a large number of cellular responses, e.g. ER stress, autophagy, and ROS production.

In type 2 diabetes, aggregation of IAPP into beta-cell toxic oligomers and ultimately amyloid fibrils is an accepted major cause of beta-cell loss (4). It has been taken for granted that islet amyloid formation does not occur in type 1 diabetes due to the lack of the producers. However, we have previously shown that, close to the onset of type 1 diabetes, a subset of patients exhibited very high plasma IAPP concentrations leading to an abnormal IAPP-to-insulin plasma ratio (5). We therefore questioned whether IAPP aggregation and islet amyloid may develop during a limited period of time in type 1 diabetes disease.

**Figure 1.**
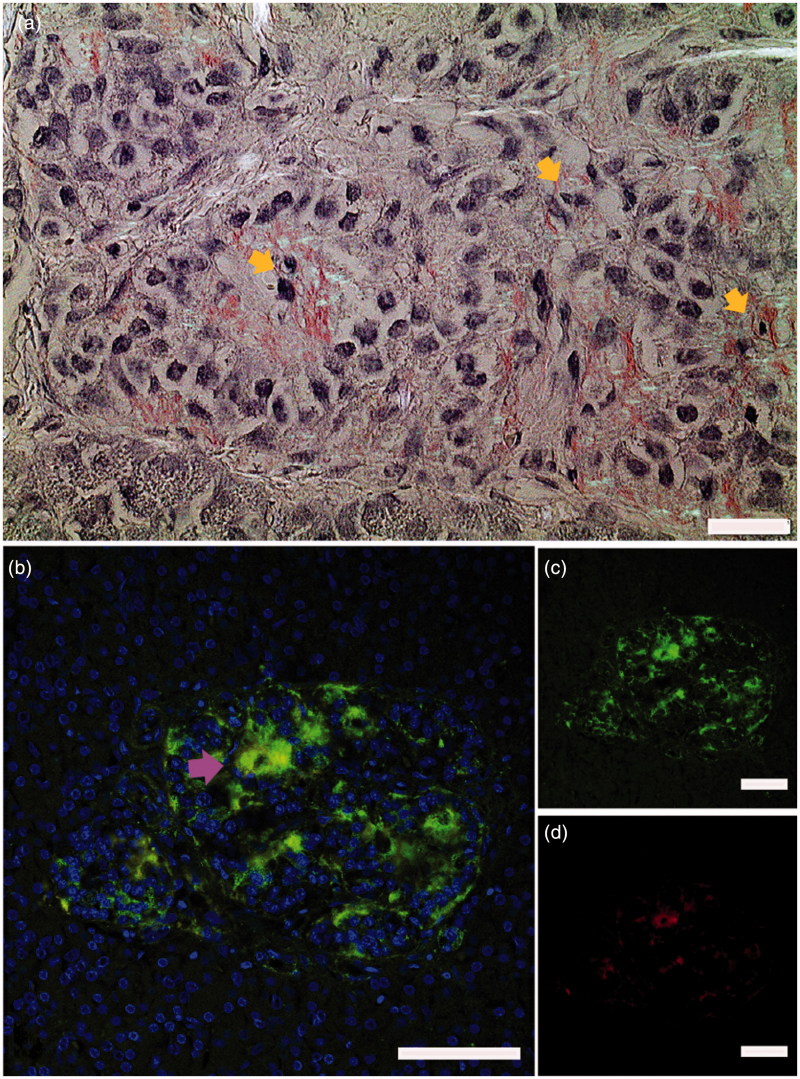
Islet amyloid was detected in pancreata obtained from patients newly diagnosed with type 1 diabetes. (a) a section from a pancreas tail biopsy stained with Congo red showing multiple islets with amyloid. Intracellular amyloid is associated with pyknotic cell nuclei indicated with yellow arrows. (b–d) IAPP was detected with a hIAPP specific antibody and visualized with an Alexa-488-labelled detection antibody (green), and amyloid by a subsequent staining with Congo red (red). Co-localization of IAPP immunoreactivity and amyloid (yellow) indicated by arrowhead in magenta ensures that amyloid is made up of IAPP. Bar 20 µm.

We studied sections of six pancreas biopsies from live adults aged 18–35 years with recent-onset type 1 diabetes included in the Diabetes Virus Detection (DiViD) study and detected islet amyloid in two patients. The DiViD study was approved by the Government’s Regional Ethics Committee (Norway), and ethical issues have been discussed previously (6,7). The biopsies were minimal pancreatic tail resections laparoscopically collected 3–9 weeks after diagnosis (6) and offer a unique opportunity to investigate early morphological changes in type 1 diabetes. One section of each biopsy was stained for amyloid and examined in a polarization microscope. In two out of the six biopsies we found islets with amyloid. In these amyloid-positive sections approximately 10% of the islets were affected. Both intra- and extracellular amyloid were detected and were seen in association with pyknotic cell nuclei, indicating dying cells (Figure 1(a)). Intracellular deposition may be an early event in islet amyloid development.

Pancreas sections from five age-matched non-diabetic individuals were stained for amyloid with Congo red, and a minimum of 50 islets from each patient were scrutinized, but no amyloid could be detected. To verify that the amyloid consisted of IAPP, sections were incubated with anti-IAPP antiserum (produced in rabbit against residues 20–29 of IAPP), and reactivity was detected with an Alexa-488-conjugated goat anti-rabbit antibody, while amyloid was detected with Congo red. Evidently, there was co-localization with amyloid in red and IAPP in green (Figure 1(b–d)).

Already many years ago, IAPP was shown to form IAPP amyloid in human islets transplanted under the kidney capsule of nude mice (8,9). IAPP amyloid also develops in human islets implanted into the liver of patients with type 1 diabetes (10). It is likely that isolation of islets and implantation to a new environment, often with higher glucose concentrations, imposes a stress state with increased requirement of hormone release that in turn leads to IAPP aggregation and amyloid formation. Interestingly, we have detected a low-grade persistent enterovirus infection in the DiViD cases (11), which may have induced beta-cell stress in some islets.

Protein aggregation, from monomer to mature amyloid fibrils, is complex and includes formation of oligomers. Such oligomers are considered cytotoxic and are linked to beta-cell death (12). We recently described high concentrations of circulating IAPP (up to 1000 pmol/L) in samples taken at the time of diagnosis of type 1 diabetes in children (5). The dramatic increase in IAPP was not accompanied by an increase in C-peptide and thus supports a dissimilar regulation of IAPP and insulin secretion. A high concentration of IAPP is believed to be a determining factor in IAPP amyloidogenesis. Plasma IAPP was, however, low at the time of biopsy in our six patients. It is possible that plasma IAPP concentrations fluctuate, or that high concentrations of IAPP occur only for a limited time, and that this peak already had passed.

Our finding of islet amyloid early in type 1 diabetes may indicate a process of beta-cell destruction caused by IAPP aggregation. Such destruction would result in exposure of beta-cell specific proteins with capacity to act as autoantigens. Therefore, we suggest that formation of cytotoxic IAPP amyloid could constitute a possible mechanism in the pathogenesis of type 1 diabetes in a sub-group of patients.
